# Supplementary material for: Association between pre-competition strength and sprint canoe/kayak performance: A mixed-effects analysis of professional Chinese athletes
Source: PLoS One. 2026 May 28;21(5):e0347178. doi: 10.1371/journal.pone.0347178 (PMC13218461; doi:10.1371/journal.pone.0347178)
Supplement: Attachment — Submitted filename: Response letter.docx [file pone.0347178.s003.docx]

**Dear Dr. Danica Janicijevic, dear reviewers,**

Please find enclosed a revision of our manuscript, *“Association between pre-competition strength and sprint canoe/kayak performance: A mixed-effects analysis of professional Chinese athletes”.* We sincerely appreciate the opportunity to revise and improve our work. We would like to extend our gratitude to the reviewers for their thoughtful and constructive comments, which have substantially strengthened the manuscript. In particular, we are grateful for the reviewers’ professional, detailed, and highly actionable suggestions regarding the statistical analyses, which greatly clarified and facilitated the revision process. Changes to the original manuscript are highlighted in red font and an itemized point-by-point response to the reviewers’ comments is presented below.

**AUTHORS’ RESPONSE TO THE REVIEWERS**

**Reviewer 1#**

**GENERAL COMMENTS:**

Thank you for the opportunity to review the manuscript titled “Is the pre-competition strength test a determinant of sprint canoe and kayak race performance? A dataset analysis of professional Chinese athletes.”

The topic is highly relevant for sports science and performance analytics in sprint canoeing and kayaking. The study’s use of real competition data from professional athletes is particularly valuable and rare. Overall, the manuscript is clearly written and well structured, demonstrating careful data collection and logical discussion.

However, to reach the level of statistical and methodological rigor expected by PLOS ONE, substantial improvement in the analytical approach is required. The main concern lies in the statistical treatment of the data, as the current use of Pearson correlations and stepwise multiple regression does not account for the hierarchical and potentially correlated structure of the dataset.

I provide below a set of constructive and detailed recommendations aimed at strengthening the analytical framework, improving transparency in the Methods section, and enhancing the interpretability of the findings. These revisions will significantly increase the robustness, reproducibility, and scientific credibility of the manuscript.

**REPLY TO GENERAL COMMENTS:**

We are grateful for the opportunity to revise and improve our work. We are grateful for your recognition of the value of our research topic and the overall presentation of the original manuscript. We acknowledge that the use of relatively simple statistical methods in the initial submission limited the strength of our conclusions, largely due to our limited background in advanced statistical analysis. Following your recommendations, we have adopted the GLMM framework and thoroughly reconstructed all statistical procedures. We also re-organized the manuscript to improve clarity and better align with your guidance. We hope that the revised version now meets your expectations and adheres to the required standards.

**SPECIFIC COMMENTS:**

**COMMENT 1:**

1. Statistical Analyses (Core recommendation – revise prior to other sections)

Major Revision Required.

The analytical strategy should be restructured using Generalized Linear Mixed Models (GLMMs) or multilevel regression models instead of stepwise linear regression. This adjustment is essential to correctly model the nested data structure (athletes within events, distances, and sexes) and to control for between-athlete variability.

Detailed recommendations:

1. Model specification:

o Dependent variable: Race time (s). Apply log transformation if residuals show skewness or heteroscedasticity.

o Fixed effects: HDL 1RM, BP 1RM, PBP 1RM, PU repetitions, modality (canoe/kayak), distance (200, 500, 1000 m), sex, and body mass (or relative strength).

o Random effects: Intercepts for athlete ID (to capture individual variability) and possibly for event/session (to account for environmental differences between heats).

o Interactions: Strength × modality, strength × distance, and strength × sex should be examined to detect discipline-specific associations.

2. Collinearity control:

o Compute variance inflation factors (VIF).

o If high multicollinearity exists among strength variables, apply Principal Component Analysis (PCA) or Partial Least Squares (PLS) to derive independent components representing upper-body and overall strength.

3. Model selection and reporting:

o Avoid stepwise selection procedures; use theory-driven models compared via AIC/BIC or likelihood-ratio tests.

o Report marginal and conditional R² (Nakagawa & Schielzeth, 2013) to quantify variance explained by fixed and random effects.

o Provide standardized β coefficients, SE, 95% CIs, and p-values for each fixed effect.

o Include full residual diagnostics and influence measures (Cook’s distance, leverage).

4. Sensitivity analyses:

o Conduct models separately by sex and by modality to examine stability of predictors.

o Compare models with absolute versus relative strength variables.

5. Visualization:

o Present marginal effects plots showing predicted race time as a function of strength variables for each distance and modality.

o Include scatterplots with fitted regression lines and confidence intervals to depict variability and trends.

6. Interpretation:

o Emphasize the magnitude and practical relevance of the coefficients rather than focusing solely on p-values.

o Discuss potential moderation effects and limitations of cross-sectional inference.
Justification:

GLMMs provide a statistically valid framework to account for repeated or grouped measures, heteroscedasticity, and random variation across individuals and events. This will substantially strengthen the study’s inferential validity and allow generalization beyond the sample tested.

**REPLY 1:**

In accordance with your recommendation, we reanalyzed the data using a GLMM framework. Specifically, we implemented the models using glmmTMB package, for the following reasons. First, race time exhibits clear hierarchical structure across distances (200 m, 500 m, and 1000 m), and glmmTMB provides greater flexibility in modeling this type of distributional heterogeneity. Second, glmmTMB demonstrated more stable convergence when fitting multiple random effects (athlete ID and heat) and when including the required interaction terms.

o We used a Gamma family with a log link, which allows the fixed effects to be interpreted as proportional changes in race time. This approach is consistent with the reviewer’s request for standardized effect interpretation.

o The fixed‐effects structure included BP 1RM, PBP 1RM, HBD 1RM, PU MNR, modality (canoe/kayak), distance (200, 500, 1000 m), sex, and body mass. For the 1RM variables, we compared models using both absolute (kg) and relative (kg/kg) strength values.

o The random-effects structure included athlete ID and heat. The dataset comprised outcomes from 28 heats, which aligns with the your recommendation to account for potential environmental or grouping effects on race time.

o We considered interaction terms of strength × modality, strength × distance, and strength × sex. Accordingly, we compared eight candidate models combining absolute vs. relative strength and the different interaction terms (strength, strength × modality, strength × distance, strength × sex). Model selection was based on AIC, VIF diagnostics, and residual checks. The final selected model was the relative strength × distance model, as it showed no high multicollinearity among strength variables, well-behaved residuals, and the lowest AIC value.

o All model parameters are fully reported, including R², standardized β coefficients, standard errors, 95% confidence intervals, and p-values for each fixed effect. Specifically, we transformed the β coefficients into percentage changes. Given that we used a Gamma family with a log link, this allows a clear interpretation of how a one-unit change in a fixed effect corresponds to a proportional (%) change in race time.

o For sensitivity analyses, we conducted separate models by sex and by modality, as recommended by you.

o Marginal effects and scatterplots with fitted regression lines were plotted for each strength variable to visualize the predicted race time and observed data.

**COMMENT 2:**

Title

After reanalyzing the data using GLMMs, the title should reflect the updated analytical rigor and sample characteristics.

Suggested revision:

“Association between pre-competition strength and sprint canoe/kayak performance: a mixed-effects analysis of professional Chinese athletes.”

This version is concise, informative, and communicates both the population and the analytical approach.

**REPLY 2:**

The title has been revised as suggestion.

**COMMENT 3:**

Abstract

The abstract is generally well structured, but the Results section must be rewritten after the new analyses. The current numerical values (r, R², etc.) will change once GLMMs are applied.

Include a sentence such as:

“Results will be updated after reanalysis using mixed-effects models that adjust for within-athlete and event-level variability.”

Additionally, explicitly mention:

• Study design (cross-sectional, observational, based on official competition data).

• Main analytical approach (mixed-effects modeling).

• Practical implications for coaches and performance monitoring.

**REPLY 3:**

The Abstract section has been revised as suggestion: *“This study aimed to examine the associations between pre-competition strength measures and sprint canoe and kayak performance. A post-hoc observational cross-sectional analysis was conducted using official results from the 2023 National Canoe Sprint Autumn Championships (China). Pre-competition strength testing included the one-repetition maximum of the hexagonal deadlift (HDL), bench press (BP), and prone bench pull (PBP), as well as the maximum number of repetitions in the pull-up (PU). Preliminary race results from single-paddler canoe and kayak events were analyzed, including men’s 200-m and 1000-m races and women’s 200-m and 500-m races. A generalized linear mixed-effects model was used to analyze the data (n = 133; 69 men, 64 women). Fixed effects included relative strength (HDL, BP, PBP, and PU), distance (200 m, 500 m, and 1000 m) and their interactions, sex (male and female), modality (canoe and kayak), and body mass. Athlete identity and heat were treated as random effects. The main findings were: (i) The main model revealed that PBP significantly affected 200 m race time (estimate = −16.7%, p < 0.001), but its effect was reduced at 500 and 1000 m (−6% to −1%); (ii) sensitivity analyses indicated that PBP had a greater influence on race time in men (−19.7% to −7.8%) than in women (−10.9% to 2.6%), whereas PU was significant only in women (−0.254%, p = 0.043); (iii) PBP had a stronger impact on race time in kayak than in canoe (−25.7% to −8.3% vs. −8.6%). Although no strength variables reached statistical significance in canoe (p = 0.060–0.239), improvements in PU may still meaningfully contribute to race performance in this modality (95% confidence interval = −0.45% to 0.00935%). Therefore, integrating targeted upper-body resistance training into pre-competition preparation may therefore support improved race outcomes in Chinese professional canoe and kayak athletes.”*

**COMMENT 4:**

Introduction

The introduction effectively contextualizes the relevance of muscular strength for canoe and kayak sprint performance. However, the rationale for the statistical design should be better integrated.

Recommendations:

• Highlight the gap that previous studies did not account for nested data structures (e.g., repeated measures across distances or modalities).

• Explicitly state testable hypotheses (e.g., “Upper-body strength variables, particularly PBP 1RM, are expected to explain greater variance in kayak than canoe performance”).

• Expand on the biomechanical and energetic differences between canoe and kayak that justify separate modeling.

• Include references supporting advanced modeling approaches in sports science (e.g., Gelman & Hill, 2020; Nakagawa & Schielzeth, 2013).

**REPLY 4:**

We appreciate your recognition of the structure of the Introduction in our original manuscript. Corresponding revisions have been made as follows:

o Statistical limitations of previous studies have been highlighted: *“From a statistical perspective, previous studies either failed to account for the nested structure of the data (e.g., repeated observations across distances or modalities) or did not incorporate random effects to control for sources of variability (e.g., individual athlete differences or grouping effects).”*

o Reasonable hypotheses have been added: *“We hypothesized that (i) upper-body strength variables, particularly PBP 1RM, are expected to have a stronger influence on kayak performance compared with canoe performance, and (ii) their influence is expected to be greater for shorter distances (200 m) than for longer distances (500 m and 1000 m) [2].”*

o Rationale for conducting a comprehensive investigation has been elaborated in more detail: *“These issues highlight the need for a comprehensive investigation conducted under official competition conditions to better understand the relationship between pre-competition strength measures and race performance, given that: (i) canoeing and kayaking differ in both biomechanics and energetic demands; (ii) different race distances rely on distinct energy system contributions; and (iii) male and female athletes display well-established differences in muscular characteristics [2].”*

o Recommended references have been added to support the study objectives.

**COMMENT 5:**

Materials and Methods

The methodology is clearly presented and well justified, yet several clarifications are needed for full reproducibility:

• Testing protocol: Provide the order of tests, rest intervals, warm-up procedures, and the number of trials allowed to reach 1RM.

• Instrumentation: Specify brands, calibration methods, and assessors’ qualifications.

• Normalization: Indicate whether strength variables were normalized to body mass; if not, consider reanalyzing both absolute and relative metrics.

• Confounders: Include age, competitive experience, or environmental variables (temperature, wind speed) as potential covariates.

• Data management: Describe handling of missing data (if any) and confirm that all athletes included completed all tests.

• Statistical section (to be rewritten): Clearly state that GLMMs were employed, identify the software and packages used (e.g., R lme4, lmerTest), and report model diagnostics and validation criteria.

These additions will make the study more transparent and replicable.

**REPLY 5:**

We sincerely appreciate your valuable suggestion. We have implemented the following revisions to make the Methods section more transparent:

o Following your recommendation, heats were included as a random effect and the data were reorganized into long format in preparation for GLMM analysis; during this process, we identified a minor error in the identification of participants in the original dataset. In the revised manuscript, the number of participants has been carefully checked and corrected, ensuring the accuracy of the study’s conclusions.

o The temperature, water temperature, wind direction, and wind speed for each race session were reported. Since official environmental measurements were only taken immediately before each race session (200 m, 500 m, 1000 m), it was not possible to include variables such as temperature or wind speed as covariates in the analysis, as you suggested. However, our newly included random effect for heat accounts for the potential influence of varying environmental conditions on race time.

o The testing protocols for 1RM and MNR have been added.

o Information regarding the equipment brands and the qualifications of technical officials has been.

o Both absolute and relative strength were considered as potential predictors, and this description has been added to the Statistical Analysis section.

o Age and competitive experience were not considered, as these details were not reported in the official competition results. We contacted the Chinese Canoe Association to obtain the data, but they indicated that the information is no longer available. Although environmental variables were recorded for only three race sessions and we attempted to include them as covariates, the models did not converge. However, our newly included random effect for heats likely accounts for the environmental conditions present in each session.

o This information has been added to the “Characteristics of the Dataset” section: athletes who did not complete all strength tests, or who participated in the race but did not undergo the strength assessments, were excluded from the current dataset.

o The “Statistical Analysis section” has been revised as suggestions: *“Statistical analyses were performed using R (version 4.4.3), and the significance level was set at α = 0.05. Descriptive data for athletes’ strength measures and race performance are presented as mean ± standard deviation (SD). The glmmTMB package was used to fit generalized linear mixed models (GLMM) with a gamma family and log link to allow assessment of effects and errors in percentage units. Race time was treated as the dependent variable, with athlete ID and heat included as random effects. Fixed effects included HDL 1RM, BP 1RM, PBP 1RM, PU MNR, sex (male vs. female), modality (C1 vs. K1), distance (200 m vs. 500 m vs. 1000 m), and body mass. Both absolute strength (kg) and relative strength (kg/kg) were considered, along with interaction terms for strength × sex, strength × modality, and strength × distance. The main model selection was based on the following criteria: (i) lowest akaike information criterion (AIC) value; (ii) variance inflation factors (VIF) for strength variables < 10; and (iii) reasonable model residuals, assessed using DHARMa simulations, including tests for residual uniformity, dispersion, and outliers [12, 14]. For the main model, marginal and conditional R2 values were reported [13], and fixed effects were presented as β coefficients expressed in percentage changes (calculated as % change = [e^β − 1)] × 100), along with their standard errors (SEs), 95% confidence intervals (CIs), and p-values. Sensitivity analyses were conducted by running the models separately for each sex and for each modality to examine the stability of the predictors. The ggplot2 package was used to visualize the marginal effects of strength measures on race time and the scatterplots of the observed data.”*

We believe these revisions address your comments. Furthermore, we have uploaded our original dataset along with the R code as supplementary materials to ensure that the study results are fully reproducible.

**COMMENT 6:**

Results

The current results are neatly organized, with well-structured tables and coherent text. However, the values and inferences must be updated following the GLMM reanalysis.

Maintain the current presentation structure, but after reanalysis:

• Replace the correlation and stepwise regression tables with mixed-model results (fixed effects, random effects variance, R², SE, CI, AIC).

• Add visualizations of predicted race times across strength levels for each distance and modality.

• Report effect sizes and interpret their practical meaning (e.g., “a 10 kg increase in PBP 1RM corresponds to a 0.8 s reduction in race time”).

**REPLY 6:**

All suggested revisions from you have been incorporated into the latest manuscript, including: (1) reporting of the main model’s AIC, R², random effects variances, and fixed effects estimates, SEs, 95%CIs, and p-values; (2) presentation of multicollinearity and residual diagnostics for the main model; (3) visualization of marginal effects and scatterplots; and (4) results of sensitivity analyses conducted separately by sex and by modality.

**COMMENT 7:**

Discussion

The discussion is well written and grounded in relevant literature, but it must be updated to integrate the findings from the mixed-effects models.

Maintain the current logical structure (comparison with literature, biomechanical rationale, and practical implications), but add:

• A reflection on how the use of GLMMs enhances result reliability by accounting for inter-individual variability and environmental factors.

• A paragraph explicitly recognizing that earlier stepwise regressions may have inflated associations due to lack of hierarchical control.

• Emphasize that GLMM findings may lead to more conservative but more reliable estimates of the true relationships.

• Discuss potential limitations of the dataset (e.g., only preliminary races, uncontrolled testing order, limited sample sizes in subgroups).

**REPLY 7:**

o The updated statistical analysis results have been incorporated into the Discussion section as follows: *“The main findings of this study were as follows: (i) PBP exerted a significant effect on race time at 200 m; however, this effect was markedly attenuated at longer distances (500–1000 m); (ii) The influence of PBP on race time was greater in men than in women, whereas PU showed a significant effect only in women; (iii) PBP had a stronger impact on race time in K1 compared with C1. Although no strength variables reached statistical significance in C1, improvements in PU may still offer practically meaningful contributions to race performance.”*

o A new paragraph discussing the methodological rationale for using GLMMs has also been added to the Discussion: *“A notable limitation of previous studies examining the effects of strength measures on sport performance is the use of stepwise linear regressions without hierarchical control, which may have inflated observed associations (e.g., Ho et al. [16], López-Plaza et al. [4], and Akca et al. [17]). Another important limitation is that individual- and group-level factors may influence performance outcomes; for example, environmental conditions may vary across heats, and individual differences among athletes may also contribute to variability in race time. These considerations highlight the necessity of using GLMM, which simultaneously accounts for strength measures and random effects associated with athletes and heats. By properly modeling the nested structure of the data, GLMM provides more conservative and reliable estimates of predictor–performance relationships, while preserving the potential to generalize findings to similar athlete populations [12-14].”*

o The third limitation in the Limitations section has been revised as follows, because the potential environmental effects are already modeled via the random-effect term for heat: “although the overall sample size (n = 133) is a advantage of this study, the limited sample sizes within specific subgroups (*i.e.,* sex, modality, and distance) may reduce the stability of the corresponding estimates, and thus these findings should be interpreted with caution.”

**COMMENT 8:**

Keep the current conclusion structure but condition it explicitly on the forthcoming reanalysis.

Suggested addition:

“These conclusions will be revisited after implementing mixed-effects modeling, which will allow for adjusted estimates that better capture within-athlete variability and event-level effects.”

The final statement should highlight that the study provides preliminary but promising evidence for the predictive role of upper-body strength in sprint canoe/kayak performance.

**REPLY 8:**

The Conclusion section has been revised as suggestion: *“Using GLMM that accounted for both individual athlete differences and heat-level variation, the present study identified muscular strength, particularly upper-body strength, as a meaningful predictor of race performance in professional Chinese sprint canoe and kayak athletes. Overall, athletes with higher PBP 1RM (expressed relative to body mass) performed better in competition, although its influence diminished as race distance increased. When considering sex, PBP 1RM was more closely related to race performance in male athletes, whereas PU MNR was more relevant in female athletes. When considering modality, PBP 1RM contributed more to kayak performance, whereas PU MNR appeared more relevant for canoe. These findings provide initial evidence derived from real competition settings that pre-competition upper-body strength may hold predictive value for sprint canoe/kayak performance. Integrating targeted upper-body resistance training into pre-competition preparation may therefore support improved race outcomes.”*

**COMMENT 9:**

The reference list is appropriate and up to date, but it lacks key methodological sources supporting modern statistical modeling. Please add:

• Gelman, A. & Hill, J. (2020). Data Analysis Using Regression and Multilevel/Hierarchical Models.

• Nakagawa, S., & Schielzeth, H. (2013). “A general and simple method for obtaining R² from generalized linear mixed-effects models.” Methods in Ecology and Evolution, 4(2), 133–142.

• Burnham, K.P., & Anderson, D.R. (2002). Model Selection and Multimodel Inference: A Practical Information-Theoretic Approach.

Including these references will reinforce the methodological justification for the proposed analytical changes.

**REPLY 9:**

Suggested references have been added.

**COMMENT 10:**

Final Comment

The revisions proposed—particularly the adoption of mixed-effects modeling, clearer methodological reporting, and restructured results presentation—will significantly improve the statistical robustness, interpretive depth, and scientific integrity of the manuscript. Once these adjustments are implemented, the study will make a strong and credible contribution to the literature on performance determinants in sprint canoeing and kayaking.

I commend the authors for their valuable dataset and encourage them to pursue these enhancements to maximize the impact of their work.

**REPLY 10:**

Thank you for the positive evaluation and constructive guidance. The manuscript has been revised accordingly, with all recommended methodological and analytical improvements incorporated. We appreciate your recognition of the study’s contribution and will continue to refine the work to ensure its scientific rigor and impact.

**Reviewer #2**

**GENERAL COMMENTS:**

Firstly, I would like to thank you for placing your trust in me as a reviewer and congratulate the authors on their study. Secondly, I would like to point out that my comments are strictly technical and in no way reflect my personal opinion of the authors or the quality of the study.

The study presented is interesting, well written, and the information contained therein has the potential to be published.

**REPLY TO GENERAL COMMENTS:**

We sincerely thank the reviewer for the positive evaluation of our work and for the constructive technical comments provided. We appreciate the acknowledgement of the study’s potential and have carefully addressed all suggestions to further strengthen the methodological rigor and clarity of the manuscript.

**SPECIFIC COMMENTS**

**COMMENT 1:**

I highlight the sample size and the athletes' qualifications as strengths, but perhaps the topic of analysis could have been approached from a different angle.

It is well known that strength is closely associated with athletic performance. This fact is independent of the nature of the sport, and there seems to be a weakening of this relationship as the volume of effort increases.

With this in mind, I ask the authors to better explain their scientific contribution with this material, clearly highlighting the original aspect of this study.

**REPLY 1:**

We appreciate your thoughtful comment and fully understand the concern. The positive association between strength and sport-specific performance is well established across many sports, including team sports (*e.g.,* basketball) and individual disciplines (*e.g.,* sprinting). Therefore, the aim of the present study was not to reiterate this general relationship, but to provide novel, sport-specific evidence based on a uniquely rigorous dataset and analytical approach.

Specifically:

(i) all strength tests were conducted through a standardized, federation-organized protocol immediately before a major national competition;

(ii) the interval between testing and actual race performance was only 1–3 days, ensuring a close match between measured strength and competitive readiness;

(iii) the dataset comprises elite professional athletes from multiple provincial teams, representing diverse training backgrounds and anthropometric characteristics; and

(iv) the use of GLMM allowed us to account for individual differences, heat-level variability, and potential collinearity issues inherent in stepwise regression (Based on reviewer 1’s comments).

Together, these features provide a level of ecological validity and analytical rigor not present in prior studies, allowing our findings to offer more precise and generalizable insights that can meaningfully inform training prescription for high-level canoe and kayak athletes.

We have added the corresponding description in the Discussion section to highlight the necessity of conducting this study: *“This study offers several novel contributions. First, strength measures were assessed 1–3 days before official competitions organized by competition authorities, offering a rare opportunity to examine performance predictors under realistic competitive conditions. Second, the sample included high-level professional athletes from multiple provinces, capturing diverse training backgrounds and demographic characteristics. Third, the use of GLMM allowed simultaneous consideration of individual differences and heat-level variability, yielding more accurate and generalizable estimates than traditional stepwise regression approaches.”*

**COMMENT 2:**

An interesting relationship to investigate is the level of strength and the ranking of athletes in the events mentioned. This would make it possible to infer whether the stAnother possible analysis would be that of the main components, where strength tests by body segment would be grouped together and their influence on the result calculated.rongest are indeed the winners.

**REPLY 2:**

We appreciate your suggestion. Considering that the dataset includes 28 heats and that athlete strength varied between heats (as heat assignments were influenced by pre-competition testing results), comparing only the winners’ race times could introduce additional variability, making interpretation less straightforward. Therefore, we retained race time as the outcome for clearer interpretation. Regarding principal component analysis, as suggested by Reviewer 1, combining strength measures by body segment can help reduce multicollinearity and improve model accuracy. However, in our GLMM, the collinearity among strength variables was within acceptable limits, so analyzing them individually allows for more practical, training-relevant recommendations.

**COMMENT 3:**

In the discussion, my suggestion is to revise the section of the article so that it focuses on explaining why there are relationships between the variables. Yes, it is interesting to list studies in which the results were similar or conflicting to yours, but explaining them is more important.

**REPLY 3:**

We appreciate your comment. Additional explanations for the observed relationships have been incorporated into the Discussion section. Specifically, we expanded the rationale behind why certain strength measures were more strongly associated with performance outcomes: *“Consistent with previous findings, upper-body muscular strength appears to be a more important determinant of race performance than lower-body strength, with PBP 1RM emerging as the most influential predictor among the strength measures [3, 4, 17]. However, the significant strength × distance interactions showed that the influence of PBP 1RM diminished as race distance increased. This pattern aligns with the physiological distinction between events: shorter races depend more on rapid force production, whereas longer distances place greater demands on metabolic efficiency [16]. Body mass also emerged as a significant predictor, which is likely related to its contribution to absolute force generation and propulsion in paddle strokes [24]. The effect of PBP differed between modalities, with a larger impact observed in kayak than in canoe. This discrepancy is consistent with the technical demands of the two modalities, as kayakers apply force bilaterally while canoeists generate propulsion unilaterally, reducing the specificity of bilateral strength tests for canoe [9, 23]. Strength measures contributed differently between sexes in the present dataset. Male athletes showed a greater dependence on PBP 1RM, whereas race performance in female athletes was more closely associated with PU MNR. Accordingly, resistance-training programmes for Chinese professional canoe and kayak athletes may need to be differentiated by sex and modality to maximise performance outcomes [21].”*

**COMMENT 4:**

Once again, thank you for the opportunity to read this study and contribute to the work of my Chinese colleagues. I hope that my comments will assist in the writing of parts of the study and that the final outcome will be the publication of the work.

Best regards!

**REPLY 4:**

We sincerely appreciate your thoughtful comments and constructive feedback. Your suggestions have been very helpful in improving both the clarity and the scientific rigor of our manuscript. We are grateful for your support of our work and hope that the revised version meets the expectations of the reviewers and the journal. Thank you again for your time and valuable contribution.
