# Supplementary material for: Association between pre-competition strength and sprint canoe/kayak performance: A mixed-effects analysis of professional Chinese athletes
Source: PLoS One. 2026 May 28;21(5):e0347178. doi: 10.1371/journal.pone.0347178 (PMC13218461; doi:10.1371/journal.pone.0347178)
Supplement: Attachment — Submitted filename: Response to reviewers.docx [file pone.0347178.s004.docx]

**Dear Dr. Rabiu Muazu Musa and dear reviewers,**

Please find enclosed a revision of our manuscript, *“Association between pre-competition strength and sprint canoe/kayak performance: A mixed-effects analysis of professional Chinese athletes”.* We sincerely appreciate the opportunity to revise and improve our work. Thank you very much for your time and the constructive feedback provided during this second round of review. We have carefully read and considered all the comments, which have undoubtedly helped to further refine and improve the quality of our manuscript. For your convenience, we have summarized and provided a point-by-point response to each of the reviewers’ comments below. The reviewers’ original comments are highlighted in bold, followed by our responses and the corresponding revisions made in the manuscript.

**AUTHORS’ RESPONSE TO THE REVIEWERS**

**COMMENT 1**

It is recommended to report Cook’s distance in the model diagnostics.

**RESPONSE 1**

Cook’s distance diagnostics have been performed. The maximum Cook’s distance across all observations is 0.075, which falls within the safe range (< 0.5). The correspondingly added parts in the manuscript are as follows:

Statistical analyses: “…(iii) satisfactory model diagnostics, which included reasonable residual behavior (assessed via *DHARMa* simulations for uniformity, dispersion, and outliers) and the absence of highly influential cases (verified by a Cook’s distance < 0.5) [12, 14, 16].”

Results: “…Case-level diagnostics confirmed the absence of influential observations (maximum Cook’s distance = 0.075) (Figure 2).”

**COMMENT 2**

It is recommended to report the relationship between strength improvements and changes in race performance (seconds).

**RESPONSE 2**

After reporting the main model, using the formula: Δtime = mean baseline time × (e^β× Δ^*^x^* − 1), we reported the changes in race time resulting from a 0.1-unit increase in relative strength for HDL, BP, and PBP, and a 1-repetition increase for PU within each subgroup (sex, modality, and distance). The correspondingly added parts in the manuscript are as follows:

Statistical analyses: “…To facilitate the practical application of these findings for coaches, the statistical estimates were further converted from proportional changes into absolute performance differences (in seconds). For a specific increment in a strength variable (Δ*x*), the expected change in race time (Δtime) was calculated using the following equation: Δtime = mean baseline time × (e^β× Δ^*^x^* − 1), where the mean baseline time represents the average race duration for each specific sub-group (*i.e.,* by sex, modality, and distance) within the current dataset.”

Results: “…To facilitate practical application for coaches, the estimated proportional changes in race time were converted into absolute performance differences (in seconds), as presented in Table 3.”

**COMMENT 3**

It is recommended to add the unavailability of athletes’ age, training experience, and detailed environmental covariates to the limitations section.

**RESPONSE 3**

We have added a fourth point in the limitations section: “Fourth, due to data availability constraints, variables such as athletes’ age, training experience, and detailed environmental covariates were not included.”

**COMMENT 4**

It is recommended to provide an explanation for the extremely high marginal and conditional R^2^ values.

**RESPONSE 4**

The corresponding explanation has been added to the fourth paragraph of the discussion: “In the current study, the exceptionally high marginal (99.5%) and conditional (99.9%) R^2^ inherently result from the model’s design: the ‘distance’ covariate absorbs the massive scale variance across events, the log-link Gamma specification captures proportional scaling, and the random effects account for individual baseline stability.”

**COMMENT 5**

It is recommended to modify the unit for relative strength.

**RESPONSE 5**

The unit for relative strength has been modified to (Relative to BM).

**COMMENT 6**

It is recommended to directly compare the findings of the present study with those reported in the existing literature (*e.g.,* Akca et al.).

**RESPONSE 6**

The corresponding modifications have been added to the fifth paragraph of the discussion: “Notably, Akca et al. [18] reported that PBP 1RM significantly correlated with 200-m and 500-m kayaking performance during simulated races, but not 1000-m performance. Our results confirm this distance-dependent attenuation in actual official pre-competition settings, explicitly capturing it through a significant strength × distance interaction.”

**COMMENT 7**

It is recommended to check the dataset and code to ensure the full reproducibility of the supplementary materials.

**RESPONSE 7**

The dataset has been carefully checked and found to be correct. Due to the addition of some new statistical methods, the R code has been updated accordingly, and we have rearranged its structure to make the analysis process sufficiently clear for the readers.

**ADDITIONAL CLARIFICATION**

While reviewing the code, we found that during the model selection process, the model with the "absolute strength × distance" interaction initially had the lowest AIC (1162.31), but was excluded due to severe multicollinearity (VIF > 10). According to the predefined criteria in the Statistical analyses section, we therefore searched for the model with the lowest AIC among the remaining seven models, which was the “relative strength × distance” model (AIC = 1164.85)—the model reported in the manuscript. We consider this reasonable, as the difference in AIC between the two is minimal (~2) and substantially lower than the AIC values of the other six models (1195.37–1201.04). All other analysis results have been checked and confirmed to remain identical to those in the previous round of revisions. We apologize for this oversight and sincerely provide this clarification to ensure a more transparent review process.

**ADDITIONAL REVISIONS**

We have corrected grammatical errors and typos throughout the manuscript, and provided some additional clarifications in the discussion section to enhance readability. For instance, “Although no strength variables reached statistical significance in C1, improvements in PU may still offer practically meaningful contributions to race performance, ***given that the upper bound of its 95% CI approaches zero***.”

**CONCLUDING REMARKS**

We hope that our responses and revisions have satisfactorily addressed the reviewers’ requirements and that the manuscript now meets the publication standards of the journal. We once again thank the editor and the reviewers for their time, effort, and valuable contributions to improving this manuscript.

Sincerely yours,

Authors
